# FAM83H is involved in stabilization of β-catenin and progression of osteosarcomas

**DOI:** 10.1186/s13046-019-1274-0

**Published:** 2019-06-18

**Authors:** Kyoung Min Kim, Usama Khamis Hussein, See-Hyoung Park, Mi Ae Kang, Young Jae Moon, Zhongkai Zhang, Yiping Song, Ho Sung Park, Jun Sang Bae, Byung-Hyun Park, Sang Hoon Ha, Woo Sung Moon, Jung Ryul Kim, Kyu Yun Jang

**Affiliations:** 1 Department of Pathology, Chonbuk National University Medical School, Research Institute of Clinical Medicine of Chonbuk National University-Biomedical Research Institute of Chonbuk National University Hospital and Research Institute for Endocrine Sciences, Jeonju, Republic of Korea; 2 Faculty of Science, Beni-Suef University, Beni-Suef, Egypt; 3 Department of Bio and Chemical Engineering, Hongik University, Sejong, Republic of Korea; 4 Department of Life Science, Gachon University, Seongnam, Republic of Korea; 5 Department of Orthopedic Surgery, Chonbuk National University Medical School, Research Institute of Clinical Medicine of Chonbuk National University-Biomedical Research Institute of Chonbuk National University Hospital and Research Institute for Endocrine Sciences, Jeonju, Republic of Korea; 6 Department of Biochemistry, Chonbuk National University Medical School, Research Institute for Endocrine Sciences, Jeonju, Republic of Korea; 7 Division of Biotechnology, Chonbuk National University, Iksan, Republic of Korea

**Keywords:** Osteosarcoma, FAM83H, β-Catenin, Prognosis, Ubiquitination

## Abstract

**Background:**

FAM83H was initially identified as a protein essential for dental enamel formation. Recent reports have shown that FAM83H is also involved in the progression of human cancers in conjunction with tumor-associated molecules, such as MYC and β-catenin. However, the role of FAM83H in sarcoma has not yet been investigated.

**Methods:**

The expression and roles of FAM83H and β-catenin were evaluated in human osteosarcomas from 34 patients and osteosarcoma cells.

**Results:**

The expression of nuclear FAM83H, cytoplasmic FAM83H, and β-catenin were significantly associated with each other and significantly associated with shorter survival of osteosarcoma patients by univariate analysis. In multivariate analysis, cytoplasmic expression of FAM83H was an independent indicator of shorter survival of osteosarcoma patients (overall survival; *P* <  0.001, relapse-free survival; *P* <  0.001). In U2OS, MG63, and KHOS/NP osteosarcoma cells, the knock-down of FAM83H decreased proliferation and invasion activity and overexpression of FAM83H increased proliferation and invasion activity. In KHOS/NP cells, knock-down of FAM83H significantly inhibited, and overexpression of FAM83H significantly increased in vivo growth of cells. In addition, the knock-down of FAM83H decreased protein expression of β-catenin, active β-catenin, cyclin D1, vimentin, and snail. Overexpression of FAM83H increased protein expression of β-catenin, active β-catenin, cyclin D1, vimentin, and snail. However, the expression of β-catenin mRNA was not significantly altered with knock-down or overexpression of FAM83H. In addition, FAM83H and β-catenin shown to directly interact via immunoprecipitation and nuclear and cytoplasmic localization of β-catenin was decreased with knock-down of FAM83H. Moreover, the ubiquitination and proteasomal degradation of β-catenin was increased with knock-down of FAM83H.

**Conclusions:**

This study suggests that FAM83H is involved in the progression of osteosarcomas via a mechanism involving the stabilization of β-catenin and the promotion of proliferation and invasiveness of osteosarcomas.

## Background

FAM83H was first discovered during extensive computational analysis of the human genomic sequence [1] and reported to be essential in dental enamel formation [2, 3]. Mutation of FAM83H is the main etiological factor for human autosomal dominant hypocalcified amelogenesis imperfecta [1, 3]. Moreover, recently, research into FAM83H has focused on its roles in the development and progression of human cancers. However, there are controversial reports for the role of FAM83H in human cancers [4–8]. Earlier reports, which used microarray analysis, showed higher FAM83H expression in ovarian cancers compared with normal ovarian tissue [7]. In addition, higher expression of the FAM83H gene is presented in the cancers of lung, breast, colon, liver, ovary, pancreas, and stomach [8]. The roles of FAM83H in the progression of human cancers involve changes in the proliferation and invasiveness of cancer cells. In colon cancer cells, overexpression of FAM83H is suggested to be involved in the progression of cancer cells by disorganizing keratin cytoskeleton structures [5, 6]. In addition, FAM83H increases proliferation of prostatic cancer cells [9], hepatocellular carcinoma cells [4], and clear cell renal cell carcinoma cells [10]. In hepatocellular carcinoma cells, activation of the MYC-FAM83H pathway increases the proliferation and invasion activity of cancer cells [4]. Moreover, higher expression of FAM83H is associated with an increased recurrence rate of prostatic cancer patients and shorter survival of uterine cancer [8], hepatocellular carcinoma [4], and clear cell renal cell carcinoma patients [11]. These findings suggest that FAM83H has a vital role in tumorigenesis and progression of human malignant tumors, and might be involved in the progression of various types of human cancers. However, the expression of FAM83H is down-regulated in astrocytoma and oligodendroglioma of the brain, and higher expression of FAM83H is associated with favorable prognosis of glioma and head and neck cancer patients [8]. Therefore, there is a possibility that the role of FAM83H might be different according to the type of cells and further study is needed.

Osteosarcoma is the most common primary malignant tumor of the bone and commonly involves metaphysis of the long bone [12, 13]. In the development of osteosarcoma, various genetic and clinical factors are involved, which complicate establishing a therapeutic strategy [14–17]. In addition, despite improvement of in survival of osteosarcomas with multi-agent chemotherapy, the survival of advanced osteosarcoma remains below 30% [17–19]. Moreover, the survival of osteosarcoma patients have not improved in the last few decades [13, 17, 19]. Therefore, new therapeutic approaches are needed to improve therapeutic efficacy in osteosarcoma patients, and we hypothesize that the FAM83H might be involved in the oncogenesis of the osteosarcoma based on the role of FAM83H in cancer progression in conjunction with MYC and the β-catenin pathway. In hepatocellular carcinomas, FAM83H is transcriptionally regulated by the oncogene MYC, and MYC-FAM83H signaling is involved in the proliferation of cells by affecting the expression of p27 and cyclin D1 [4]. FAM83H is also involved in the expression of p27 and cyclin D1 in clear cell renal cell carcinoma cells [20]. Therefore, when considering p27 and cyclin D1 as down-stream signaling molecules of the β-catenin pathway, there might be an association between FAM83H and the β-catenin pathway. In addition, the expression of β-catenin and cyclin D1 is associated with progression and shorter survival of patients with soft-tissue sarcomas [21]. Therefore, FAM83H and the β-catenin pathway might have roles in the progression of osteosarcomas. However, there has not been study on the role of FAM83H in malignant mesenchymal tumors such as soft-tissue sarcoma and osteosarcoma. Based on this rationale, we investigated the role of FAM83H and β-catenin in human osteosarcomas and osteosarcoma cells.

## Methods

### Osteosarcoma patients and tissue samples

In this study, 34 patients who underwent surgical resection for the primary osteosarcoma of the bone at the Chonbuk National University Hospital between January 1998 and December 2012 were evaluated. The cases included in this study consisted of the medical record, original histologic slides, and paraffin-embedded tissue blocks. All cases were reviewed according to the 2013 World Health Organization classification of tumors of soft tissue and bone [12] and the American Joint Committee on Cancer staging system [22]. The osteosarcomas were grouped according to age (< 30 y versus ≥ 30 y), sex (male versus female), tumor size (≤ 8 cm versus > 8 cm), tumor stage (I versus II-IV), histologic grade (1 and 2 versus 3 and 4), lymph node metastasis (absence versus presence), and distant metastasis (absence versus presence). The clinical information was obtained by reviewing medical records of the patients. This study was approved by institutional review board approval from Chonbuk National University Hospital and the requirement for informed consent was waved (IRB number, CUH 2016–07–023-001).

### Immunohistochemical staining and scoring in the tissue microarray

The tissue microarray (TMA) was preformed from solid areas composed mainly of tumor cells with no necrosis or degenerative changes. The diameter of each TMA core was 3.0 mm, and two cores were evaluated from each case. The TMA sections were deparaffinized, and antigen retrieval was performed by boiling the TMA tissue sections using a microwave oven for 20 min in 10 μmol/L citrate buffer (pH 6.0). Thereafter, endogenous peroxidase was blocked and incubated with Protein Block Serum-Free (DAKO, Carpinteria, CA) to prevent nonspecific staining. The tissue sections were incubated with anti-FAM83H (1:100, Bethyl Laboratories, Montgomery, TX) and anti-β-catenin (1:100, BD Biosciences, San Jose, CA) antibodies for overnight at 4 °C. The tissue sections were incubated with dextran polymers conjugated with horseradish peroxidase in DAKO Envision system (DAKO, Carpinteria, CA) and visualized with the enzyme substrate 3-amino-9-ethylcarbazole. The immunohistochemical staining slides were scored by two pathologists (KYJ and KMK) with consensus under a multi-viewing microscope without clinical information. The nuclear and cytoplasmic expression of FAM83H were separately evaluated, and the expression of β-catenin was evaluated by overall cellular level. Immunohistochemical expression of cytoplasmic FAM83H (Cy-FAM83H), nuclear FAM83H (Nu-FAM83H), and β-catenin were scored by assessing staining intensity and staining area. The staining intensity was graded from zero to three (0; no expression, 1; mild expression, 2; moderate expression, and 3; strong expression) and the staining area was graded from zero to five (0; no stained cells, 1; 1% of the cells stained positive, 2; 2–10% of the cells stained positive, 3; 11–33% of the cells stained positive, 4; 34–66% of the cells stained positive, and 5; 67–100% of the cells stained positive) [20, 23, 24]. The immunohistochemical staining score was obtained by adding the staining intensity score and area score. After that, the immunohistochemical staining score obtained from each TMA core was added. Therefore, because we have used two TMA cores in each case, the final immunohistochemical staining score for Cy-FAM83H, Nu-FAM83H, and β-catenin was ranged from zero to sixteen.

### Normal human osteoblast cells and human osteosarcoma cell lines

This study used one normal human osteoblast and three human osteosarcoma cell lines. Normal human osteoblast cells from the femoral head (PromoCell, Heidelberg, Germany) were kindly provided by Ji Hyun Park (Division of Endocrinology and Metabolism, Department of Internal Medicine, Chonbuk National University) [25]. U2OS (wild-type p53) and MG63 (mutant p53) human osteosarcoma cells were purchased from the Korean Cell Line Bank (KCLB, Seoul, Korea). KHOS/NP human osteosarcoma cells were kindly provided by Chang-Bae Kong (Department of Orthopedic Surgery, Korea Institute of Radiological and Medical Sciences) and grown in α-MEM culture media supplemented by 10% inactivated fetal bovine serum (Gibco BRL, Gaithersburg, MD) with penicillin and streptomycin (100 U/ml) [26]. The other cells were cultured in DMEM containing penicillin and streptomycin (100 U/ml) and 10% fetal bovine serum (Gibco BRL, Gaithersburg, MD) at 37 °C in a humidified incubator with 5% CO_2_.

### Transfection

The shRNA expression vectors for FAM83H were purchased from GenePharma (Shanghai, China). The sense and antisense sequences of FAM83H #1 duplex were 5′-CACCGCTCATCTTCAGCACGTCACATTCAAGAGATGTGACGTGCTGAAGATGAGCTTTTTTG-3′ and 5′-GATCCAAAAAAGCTCATCTTCAGCACGTCACATCTCTTGAATGTGACGTGCTGAAGATGAGC-3′, respectively. The sense and antisense sequences of FAM83H #2 duplex were 5′-CACCGCCGCCTCACTACAAAGAGTATTCAAGAGATACTCTTTGTAGAGGCGGCTTTTTTG-3′ AND 5′-GATCCAAAAAAGCCGCCTCACTACAAAGAGTATCTCTTGAATACTCTTTGTAGTGAGGCGGC-3′, respectively. The vector for wild-type FAM83H (Catalog #, EX-Y4473-M03; accession #, NM_198488) was purchased from GeneCopoeia (Rockville, MD). The transfection was performed using JetPRIME transfection reagent (Polyplus Transfection, Illkirch, France).

### Cell proliferation assay

The proliferation of cells was evaluated with the 3-(4,5-dimethylthiazol-2-yl)-2,5-diphenyltetrazolium bromide (MTT) cell proliferation assay (Sigma, St. Louis, MO) and colony-forming assay. For the MTT assay, 3 × 10^3^ cells were seeded in culture plates and optical density measured at a wavelength of 560 nm after 24, 48, and 72 h. The colony-forming ability of tumor cells was evaluated by growing tumor cells for 10 days in 6-well culture plates after seeding with 1 × 10^3^ cells. The number of colonies was determined using the densitometric software Clono-Counter [27].

### In vitro migration and invasion assays

The migration and invasion activity of osteosarcoma cells was evaluated by migration and invasion assays. For the migration assay, 1 × 10^5^ cells were seeded in a 24-transwell migration chamber with 8 μm-pore filters (BD Biosciences, San Jose, CA). The bottom chamber contained 10% FBS to promote migration of osteosarcoma cells. The invasion assay was performed by seeding 2 × 10^5^ cells in an 8 μm-pore Matrigel Invasion Chamber (BD Biosciences, San Jose, CA). The lower chamber in the invasion assay contained DMEM culture media with 10% FBS. The chambers for the migration and invasion assays were incubated at 37 °C for 24 h. The filters in the migration and invasion assays were stained with DIFF-Quik staining solutions (Sysmex, Japan), and the number of migrated or invaded cells was counted in five microscopic fields (magnification × 200) per well.

### Orthotopic tumorigenic model

To establish orthotopic xenografted osteosarcoma, we used six-week-old male BALB/c nude mice (Orient Bio, Seongnam, Korea). Under anesthesia, 2 × 10^6^ KHOS/NP cells transfected with control vectors, shRNA #2 for FAM83H, or the vector of wild-type FAM83H were injected into the bone marrow of the right proximal tibia. Tumor volume was calculated as length x width x height × 0.52 every week. Six weeks after tumor cell inoculation, animals were euthanized and evaluated for tumors and changes to internal organs such as lung, kidney, and liver. Animal experiments were performed with the approval of the institutional animal care and use committee of Chonbuk National University (approval number: CBNU 2019–034).

### Western blotting and immunoprecipitation

Protein extraction was performed by using phosphatase inhibitor cocktails 2, 3 (Sigma, St. Louis, MO) containing PRO-PREP Protein Extraction Solution (iNtRON Biotechnology, Korea). The lysates were centrifuged at 14,000 rpm for 10 min at 4 °C, and the protein concentration was measured by the Bradford method (Bio-Rad, Richmond, CA). The proteins were electrophoresed on SDS-polyacrylamide gel and electrotransferred to a polyvinylidene difluoride membrane. The membrane was blocked with 5% nonfat dry milk in Tris-buffered saline and 0.1% Tween 20, and incubated with primary antibodies for FAM83H (Bethyl Laboratories, Montgomery, TX), β-catenin (BD Biosciences, San Jose, CA), active β-catenin (dephosphorylated β-catenin) (Millipore, Darmstadt, Germany), cyclin D1 (Santa Cruz Biotechnology, Santa Cruz, CA), p27 (Santa Cruz Biotechnology, Santa Cruz, CA), vimentin (Santa Cruz Biotechnology, Santa Cruz, CA), snail (Abcam, Cambridge, UK), lamin B1 (Bioworld Technology, St. Louis Park, MN), and actin (Sigma, St. Louis, MO). The membranes were incubated with corresponding secondary antibodies and detected with a luminescent image analyzer (LAS-3000, Fuji Film, Tokyo, Japan) with the ECL detection system (Amersham Biosciences, Buckinghamshire, UK). Immunoprecipitation was performed with a Dynabeads-Protein A kit (Invitrogen, Carlsbad, CA). For immunoprecipitation, anti-FAM83H or anti-β-catenin antibodies were cross-linked to Dynabeads-Protein A for 20 min and incubated with protein lysates for 2 h at 4 °C. FAM83H- and β-catenin-immunoprecipitation complexes were washed thrice with supplied washing buffer and eluted by boiling in the elution buffer. Thereafter, the eluted proteins were immunoblotted with anti-FAM83H, anti-β-catenin, anti-ubiquitin, and anti-actin antibodies.

### Quantitative reverse-transcription polymerase chain reaction

An RNeasy Mini Kit (Qiagen Sciences, Valencia, CA) was used to obtain RNA and TaqMan Reverse Transcription Reagents (Applied Biosystems, Foster City, CA) were used for reverse transcription of 1.5 μg RNA. Thereafter, a quantitative reverse-transcription polymerase chain reaction was performed using Applied Biosystems Prism 7900HT Sequence Detection System and Sybr Green polymerase chain reaction Master Mix (Applied Biosystems, Foster City, CA). The expression level of the glyceraldehyde-3-phosphate dehydrogenase reference housekeeping gene was used to normalize all results and the experiments were performed in triplicate. Primer sequences for quantitative reverse-transcription polymerase chain reaction are listed in Table 1.

**Table 1:** Primer Sequences Used for Quantitative Real-time Polymerase Chain Reaction

Gene	Primer Sequence		Product size	Accession number
*FAM83H*	forward	CATGGTCCAGACAACCTGTG	214	NM_198488.3
	reverse	GCTGGATACCAGGAGGACAA		
*CTNNB1* (β-catenin)	forward	AAAATGGCAGTGCGTTTAG	100	NM_001904.3
	reverse	TTTGAAGGCAGTCTGTCGTA		
*CCND1* (Cyclin D1)	forward	GAGGAAGAGGAGGAGGAGGA	236	NM_053056.2
	reverse	GAGATGGAAGGGGGAAAGAG		
*p27*	forward	TCTACTGCGTGGCTTGTCAG	240	AB001740.1
	reverse	CTGTATTTGGAGGCACAGCA		
*Vimentin*	forward	GAGAACTTTGCCGTTGAAGC	170	NM_003380.3
	reverse	TCCAGCAGCTTCCTGTAGGT		
*SNAL1* (Snail)	forward	ACCCCACATCCTTCTCACTG	217	NM_005985.3
	reverse	TACAAAAACCCACGCAGACA		
*GAPDH*	forward	AACAGCGACACCCACTCCTC	258	NM_001256799.1
	reverse	GGAGGGGAGATTCAGTGTGGT		

Web link to accession numbers: https://www.ncbi.nlm.nih.gov/gene

### Ubiquitin proteasomal degradation and ubiquitination analysis

MG63 cells (5 × 10^5^) were seeded in 6-cm dishes and transfected with control or FAM83H shRNA. 48 h after transfection, the cells were treated with 20 μg/ml cycloheximide (CHX, Sigma, St. Louis, MO) or 20 μM MG132 (Sigma, St. Louis, MO) for 0.5 to 2 h. Western blot was performed for anti-β-catenin (Santa Cruz Biotechnology, Santa Cruz, CA) and anti-actin (Sigma, St. Louis, MO) antibodies with the cell lysates. In addition, MG63 cells transfected with control or FAM83H shRNA for 48 h were treated with 20 μM MG132 for 4 h. Thereafter, the total lysates were immunoprecipitated with anti-β-catenin antibody and blotted with anti-ubiquitin and anti-β-catenin antibodies.

### Statistical analysis

To determine positivity for the immunohistochemical staining for FAM83H and β-catenin, receiver operating characteristic curve analysis was preformed, and the cut-off points were determined at the points with the highest positive likelihood ratio point for the estimation of the death of osteosarcoma patients [15, 20, 28]. The survival analysis performed for the overall survival (OS) and relapse-free survival (RFS) of osteosarcoma patients and end point of follow-up was June 2013. For the OS analysis, the duration from the date of diagnosis to the date of death by osteosarcoma or last follow up was evaluated. An event in OS analysis was the death of patients from osteosarcoma, and the patients who were alive at last contact or death by other causes were censored for OS analysis. In RFS analysis, relapse of any type and death from osteosarcoma was an event, and the duration from the date of diagnosis to the date of the event was evaluated. The patients who were alive at last contact without relapse or death from other causes were censored for RFS analysis. The prognostic impact was evaluated by univariate and multivariate Cox proportional hazards regression analyses and Kaplan-Meier survival analysis. To compare results between the studied groups, Pearson’s χ2 test and the Student’s *t*-test were used. All experiments were done in triplicate, and the representative data are presented. SPSS software (IBM, version 20.0, CA) was used throughout and *P* value less than 0.05 was considered statistically significant.

## Results

### The expression of FAM83H and β-catenin are associated with progressed clinicopathological factors of osteosarcoma patients

**Fig. 1:**
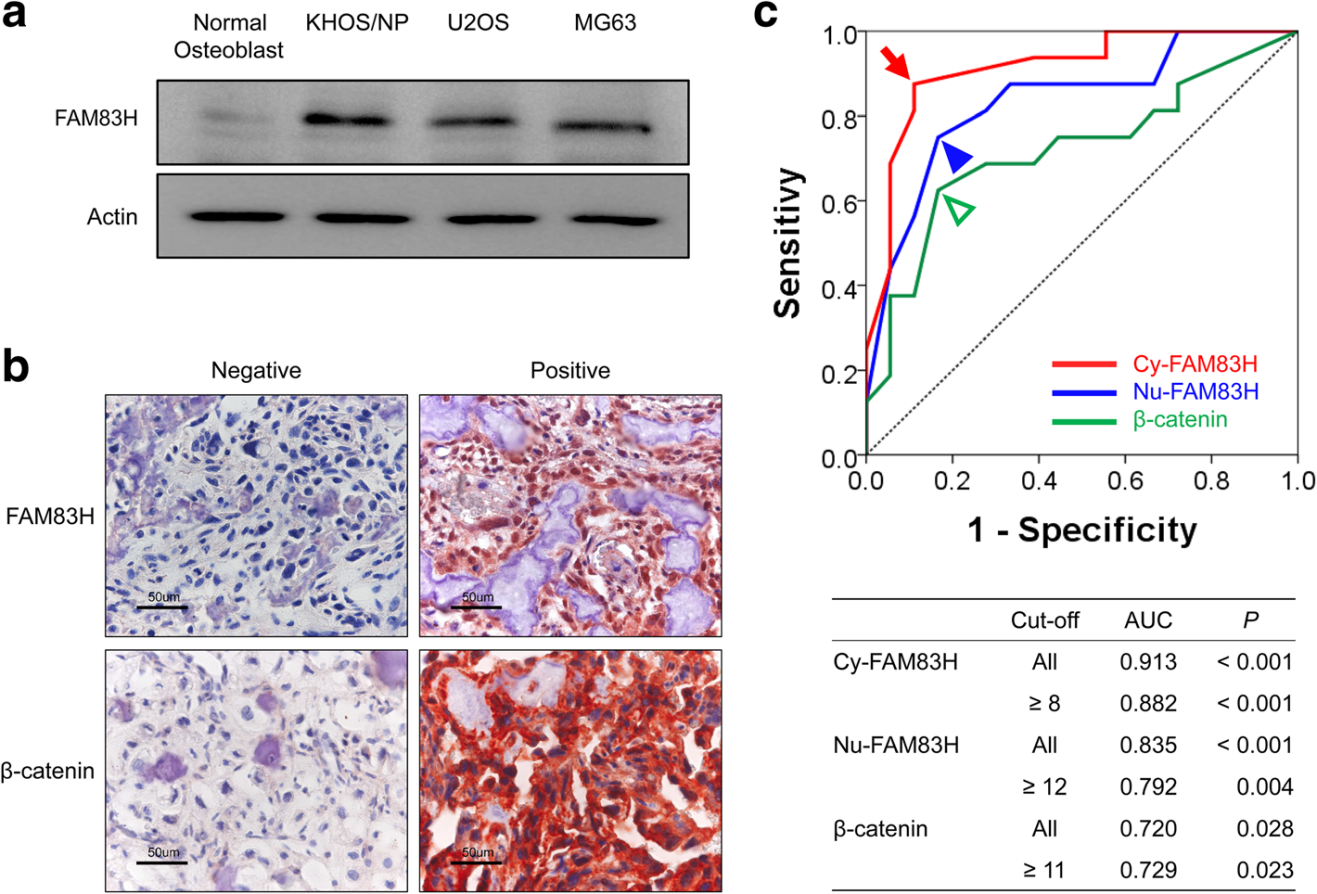
Immunohistochemical expression of FAM83H and β-catenin in human osteosarcoma tissue and statistical analysis to determine cut-off values. **a** The expression of FAM83H protein by western blot in normal osteoblast cells and U2OS, MG63, and KHOS/NP osteosarcoma cells. **b** Immunohistochemical expression of FAM83H and β-catenin in human osteosarcomas. Original magnification, × 400. **c** Receiver operating characteristic curve of cytoplasmic and nuclear FAM83H expression for death from osteosarcoma. The cut-off points for the cytoplasmic expression of FAM83H (Cy-FAM83H), nuclear expression of FAM83H (Nu-FAM83H), and β-catenin expression were selected at the highest positive likelihood ratio points for the estimation of death in osteosarcoma patients with receiver operating characteristic curve analysis. The red arrow, blue arrowhead, and green empty arrowhead indicate cut-off points for the expression of Cy-FAM83H, Nu-FAM83H, and β-catenin, respectively. AUC; area under the curve

When we compared FAM83H protein expression in normal human osteoblast cells and human osteosarcoma cells, U2OS, MG63, and KHOS/NP osteosarcoma cells showed higher expression of FAM83H compared with normal osteoblast cells (Fig. 1a). In human osteosarcoma tissue, immunohistochemical expression of FAM83H and β-catenin were observed in both the cytoplasm and nuclei of osteosarcoma cells (Fig. 1b). Although, previous reports have presented very rare expression of FAM83H in the nuclei of cells [5, 6, 29], cytoplasmic and nuclear expression of FAM83H have been presented in human cancers [4, 10]. Therefore, we evaluated FAM83H expression in the cytoplasm and nuclear expression separately. The expression of β-catenin was evaluated by its overall cellular expression. The cut-off values for the positivity of cytoplasmic expression of FAM83H (Cy-FAM83H), nuclear expression of FAM83H (Nu-FAM83H), and β-catenin expression were determined with receiver operating character curve analysis to predict the death of osteosarcoma patients. The cut-off points for the expression of Cy-FAM83H, Nu-FAM83H, and β-catenin were eight, twelve, and eleven, respectively (Fig. 1c). With these cut-off values, the positive expression of Cy-FAM83H, Nu-FAM83H, and β-catenin were seen in 47.1% (16 of 34), 44.1% (15 of 34), and 38.2% (13 of 34) of osteosarcomas, respectively. Cy-FAM83H positivity was significantly associated with age of the patients (*P* = 0.013), larger tumor size (*P =* 0.039), higher tumor stage (*P* = 0.002), and higher histologic grade (*P* = 0.002). Nu-FAM83H positivity was significantly associated with larger tumor size (*P* = 0.016), higher tumor stage (*P* <  0.001), and higher histologic grade (*P* <  0.001). In addition, there was significant association between Cy-FAM83H, Nu-FAM83H, and β-catenin expression (Cy-FAM83H versus Nu-FAM83H; *P* <  0.001, Cy-FAM83H versus β-catenin; *P* = 0.042, Nu-FAM83H versus β-catenin; *P* = 0.002) (Table 2).

**Table 2:** Clinicopathologic variables and the expression of FAM83H and β-catenin in osteosarcomas

Characteristics	No.	Cy-FAM83H		Nu-FAM83H		β-catenin	
		Positive	*P*	Positive	*P*	Positive	*P*
Age, years							
< 30	24	8 (33%)	0.013	7 (29%)	0.07	8 (33%)	0.362
≥ 30	10	8 (80%)		8 (80%)		5 (50%)	
Sex							
Male	24	13 (54%)	0.198	13 (54%)	0.068	10 (42%)	0.524
Female	10	3 (30%)		2 (20%)		3 (30%)	
Tumor size							
≤ 8 cm	17	5 (29%)	0.039	4 (24%)	0.016	6 (35%)	0.724
> 8 cm	17	11 (65%)		11 (65%)		7 (41%)	
Tumor stage							
I	11	1 (9%)	0.002	0 (0%)	< 0.001	2 (18%)	0.096
II, III, IV	23	15 (65%)		15 (65%)		11 (48%)	
Histologic grade							
1, 2	11	1 (9%)	0.002	0 (0%)	< 0.001	2 (18%)	0.096
3, 4	23	15 (65%)		15 (65%)		11 (48%)	
Lymph node metastasis							
Absence	33	15 (46%)	0.282	14 (42%)	0.253	12 (36%)	0.197
Presence	1	1 (100%)		1 (100%)		1 (100%)	
Distant metastasis							
Absence	29	12 (41%)	0.110	11 (38%)	0.080	10 (34%)	0.278
Presence	5	4 (80%)		4 (80%)		3 (60%)	
β-catenin							
Negative	21	7 (33%)	0.042	5 (24%)	0.002		
Positive	13	9 (69%)		10 (77%)			
FAM83H, nuclear							
Negative	19	3 (16%)	< 0.001				
Positive	15	13 (87%)					

Abbreviations: *Cy-FAM83H* cytoplasmic expression of FAM83H, *Nu-FAM83H* nuclear expression of FAM83H

### The expression of cy-FAM83H, nu-FAM83H, and β-catenin are significantly associated with shorter survival of osteosarcoma patients

**Fig. 2:**
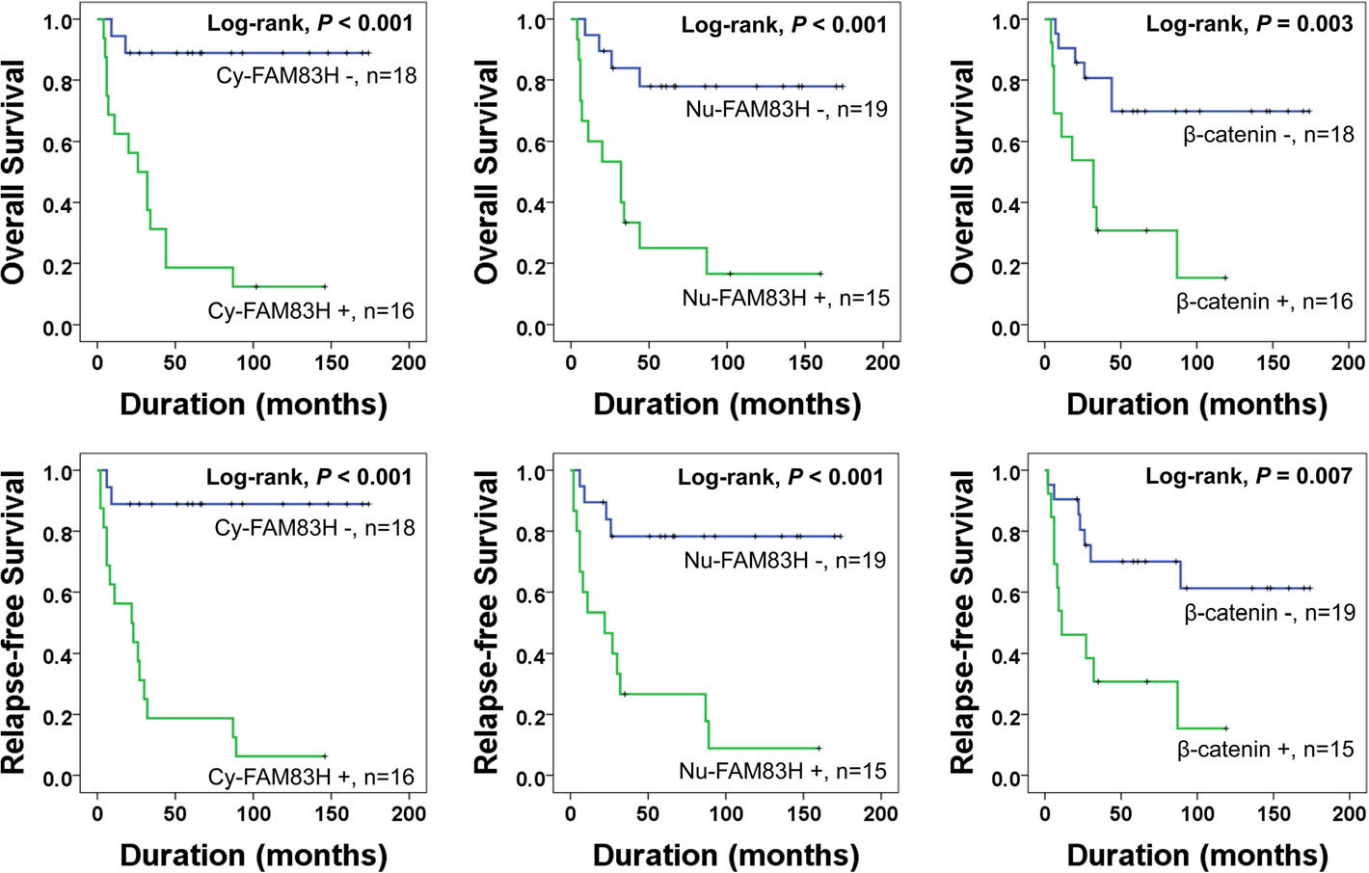
Kaplan-Meier survival analysis according to the expression of FAM83H and β-catenin in 34 osteosarcoma patients. Kaplan-Meier survival curves for the overall survival and relapse-free survival according to the cytoplasmic expression of FAM83H (Cy-FAM83H), nuclear expression of FAM83H (Nu-FAM83H), and the expression of β-catenin in osteosarcoma patients

In univariate survival analysis, age of the patients (OS; *P* = 0.048, RFS; *P* = 0.027), tumor size (OS; *P* = 0.012, RFS; *P* = 0.012), tumor stage (OS; *P* = 0.023, RFS; *P* = 0.020), histologic grade (OS; *P* = 0.023, RFS; *P* = 0.020), distant metastasis (OS; *P* = 0.006, RFS; *P* = 0.009), Cy-FAM83H expression (OS; *P* = 0.001, RFS; *P* <  0.001), Nu-FAM83H expression (OS; *P* = 0.002, RFS; *P* = 0.001), and β-catenin (OS; *P* = 0.006, RFS; *P* = 0.012) were significantly associated with both OS and RFS of osteosarcoma patients (Table 3). Cy-FAM83H positivity predicted a 12.106-fold (95% confidence interval [95% CI]; 2.736–53.563) greater risk of shorter OS and a 14.253-fold (95% CI; 3.225–62.994) greater risk of shorter RFS in osteosarcoma patients. Nu-FAM83H positivity predicted a 5.865-fold (95% CI; 1.874–18.355) greater risk of shorter OS and a 6.294-fold (95% CI; 2.038–19.431) greater risk of shorter RFS in osteosarcoma patients. The patients with a tumor of β-catenin positive had a 4.225 (95% CI; 1.514–11.788) greater risk of shorter OS and a 3.548-fold (95% CI; 1.328–9.485) greater risk of shorter RFS of osteosarcoma patients (Table 3). The Kaplan-Meier survival curves according to the expressions of Cy-FAM83H, Nu-FAM83H, and β-catenin are presented in Fig. 2.

**Table 3:** Univariate Cox proportional hazards regression analysis for the overall survival and relapse-free survival of osteosarcoma patients

Characteristics	No.	OS		RFS	
		HR (95% CI)	*P*	HR (95% CI)	*P*
Age, years, ≥ 30 (vs. < 30)	10/34	2.730 (1.01–7.377)	0.048	2.959 (1.134–7.701)	0.027
Sex, male (vs. female)	24/34	1.900 (0.537–6.719)	0.320	2.223 (0.629–7.857)	0.215
Tumor size, ≥ 8 cm (vs. < 8 cm)	17/34	4.301 (1.369–13.508)	0.012	3.901 (1.351–11.260)	0.012
Stage, ≥ II (vs. I)	23/34	10.462 (1.377–79.476)	0.023	11.154 (1.473–84.440)	0.020
Histologic grade, 3, 4 (vs. 1, 2)	23/34	10.462 (1.377–79.476)	0.023	11.154 (1.473–84.440)	0.020
Lymph node metastasis, presence (vs. absence)	1/34	10.578 (1.100–101.706)	0.041	6.224 (0.727–53.287)	0.095
Distant metastasis, presence (vs. absence)	5/34	5.324 (1.633–17.355)	0.006	4.868 (1.495–15.853)	0.009
Cy-FAM83H, positive (vs. negative)	16/34	12.106 (2.736–53.563)	0.001	14.253 (3.225–62.994)	< 0.001
Nu-FAM83H, positive (vs. negative)	15/34	5.865 (1.874–18.355)	0.002	6.294 (2.038–19.431)	0.001
β-catenin, positive (vs. negative)	13/34	4.225 (1.514–11.788)	0.006	3.548 (1.328–9.485)	0.012

Abbreviations: *Cy-FAM83H* cytoplasmic expression of FAM83H, *Nu-FAM83H* nuclear expression of FAM83H, *HR* hazard ratio, *95% CI* 95% confidence interval

Multivariate analysis was performed with the factors significantly associated with OS or RFS by univariate analysis: the age of patients, tumor size, tumor stage, lymph node metastasis, distant metastasis, histologic grade, Cy-FAM83H expression, Nu-FAM83H expression, and β-catenin expression. Multivariate analysis revealed presence of distant metastasis (OS; *P* = 0.005, RFS; *P* = 0.012) and Cy-FAM83H expression (OS; *P* <  0.001, RFS; *P* <  0.001) as independent indicators of poor prognostic of OS and RFS in osteosarcoma patients. Cy-FAM83H expression indicated a 15.463-fold (95% CI; 3.196–74.809) greater risk of death and a 15.825-fold (95% CI; 3.461–72.358) greater risk of relapse or death of osteosarcoma patients (Table 4).

**Table 4:** Multivariate Cox regression analysis for the overall survival and relapse-free survival of osteosarcoma patients

Characteristics	OS		RFS	
	HR (95% CI)	*P*	HR (95% CI)	*P*
Distant metastasis, presence (vs. absence)	9.669 (1.994–46.879)	0.005	6.577 (1.524–28.377)	0.012
Cy-FAM83H, positive (vs. negative)	15.463 (3.196–74.809)	< 0.001	15.825 (3.461–72.358)	< 0.001

Abbreviations: *Cy-FAM83H* cytoplasmic expression of FAM83H, *HR* hazard ratio, *95% CI* 95% confidence interval. The multivariate analysis was adjusted for age, tumor size, stage, histologic grade, lymph node metastasis, distant metastasis, nuclear FAM83H expression, cytoplasmic FAM83H expression, and β-catenin expression. *HR* hazard ratio, *OS* overall survival, *RFS* relapse-free survival

### FAM83H is involved in the proliferation and invasiveness of osteosarcoma cells

**Fig. 3:**
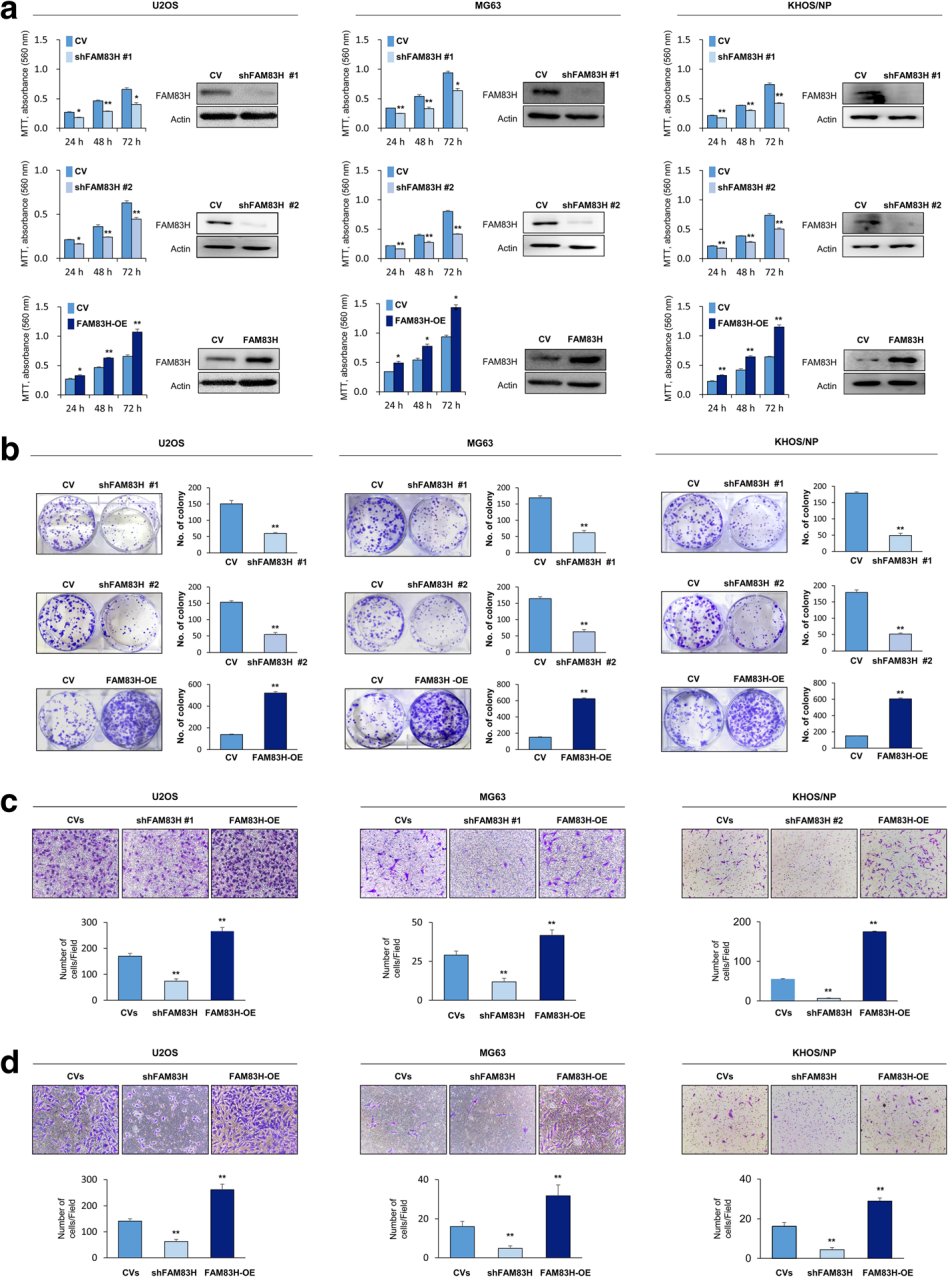
The effects of FAM83H expression on proliferation and invasiveness in osteosarcoma cells. The MTT (**a**) and colony forming assay (**b**) was performed after knock-down of FAM83H with shRNA for FAM83H #1 and #2 or overexpression of FAM83H in the U2OS, MG63, and KHOS/NP cells. The knock-down and overexpression of FAM83H after transfection is presented with western blot for FAM83H. The migration (**b**) and invasion activity (**c**) was performed after knock-down of FAM83H or overexpression of FAM83H in the U2OS and MG63 cells. CV; control vector, OE; overexpression, *; versus control vector transfection; *P <* 0.05, **; versus control vector transfection; *P <* 0.001

**Fig. 4:**
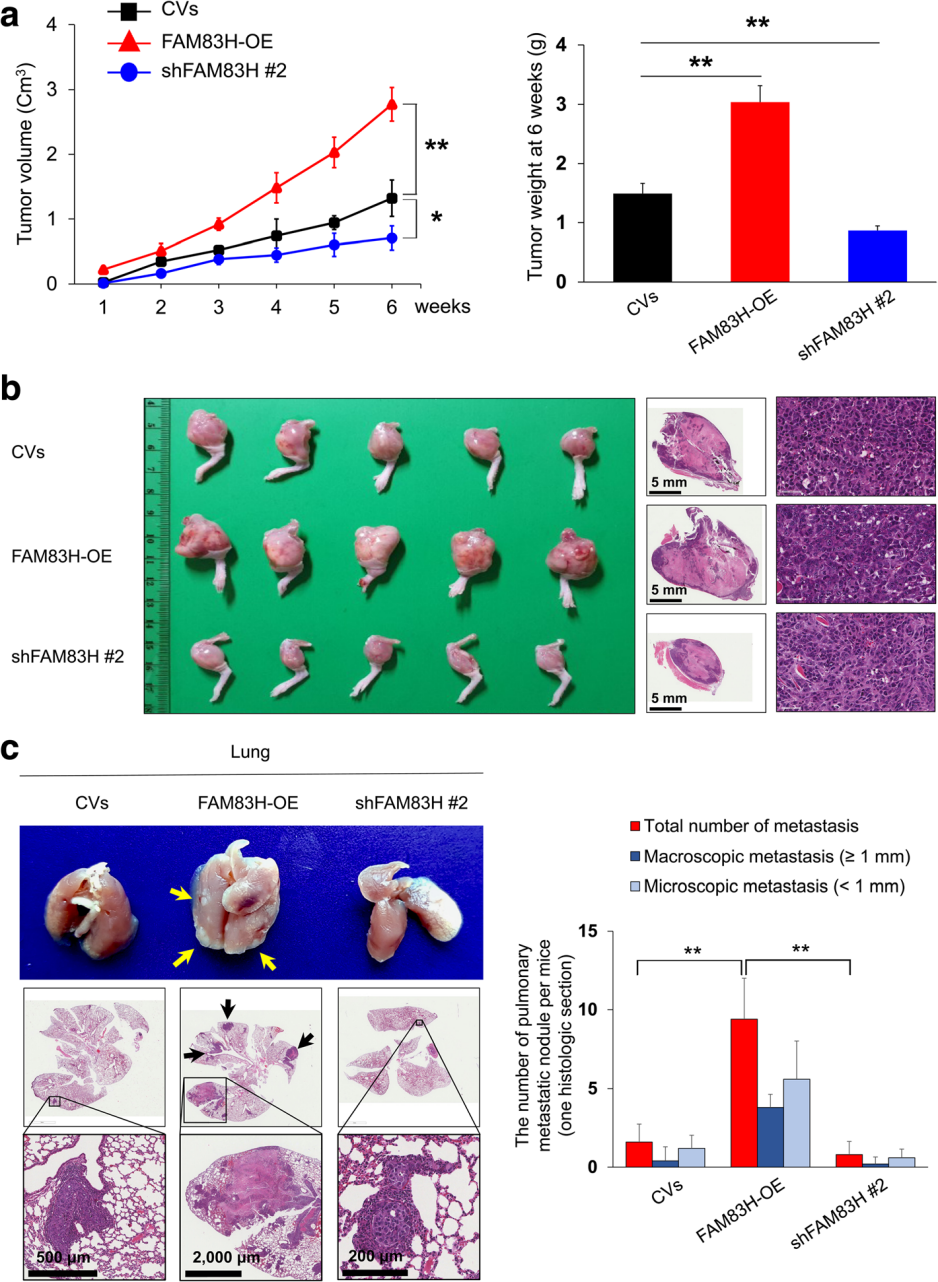
FAM83H is involved in the growth of KHOS/NP osteosarcoma cells in mice. **a** The KHOS/NP cells were transfected with control vectors, shRNA #2 for FAM83H (shFAM83H #2), or plasmid for wild-type FAM83H (FAM83H-OE) and 2 × 10^6^ KHOS/NP osteosarcoma cells were injected into the proximal tibia. The tumor volume was calculated as length x width x height × 0.52 every week and the tumor weight measured six weeks after injection of KHOS/NP osteosarcoma cells. **b** Macroscopic and microscopic findings of tumors formed around the tibia at six weeks after injection of KHOS/NP osteosarcoma cells. **c** Macroscopic and microscopic findings of pulmonary metastatic nodules of KHOS/NP cells at six weeks after injection of cells in the proximal tibia. In addition to the total number of pulmonary metastatic nodules, nodules equal to or larger than 1 mm or smaller than 1 mm were counted separately. Arrows indicate metastatic nodules. CV; control vector, OE; overexpression, *; versus control vector transfection; *P <* 0.05, **; versus control vector transfection; *P <* 0.001

**Fig. 5:**
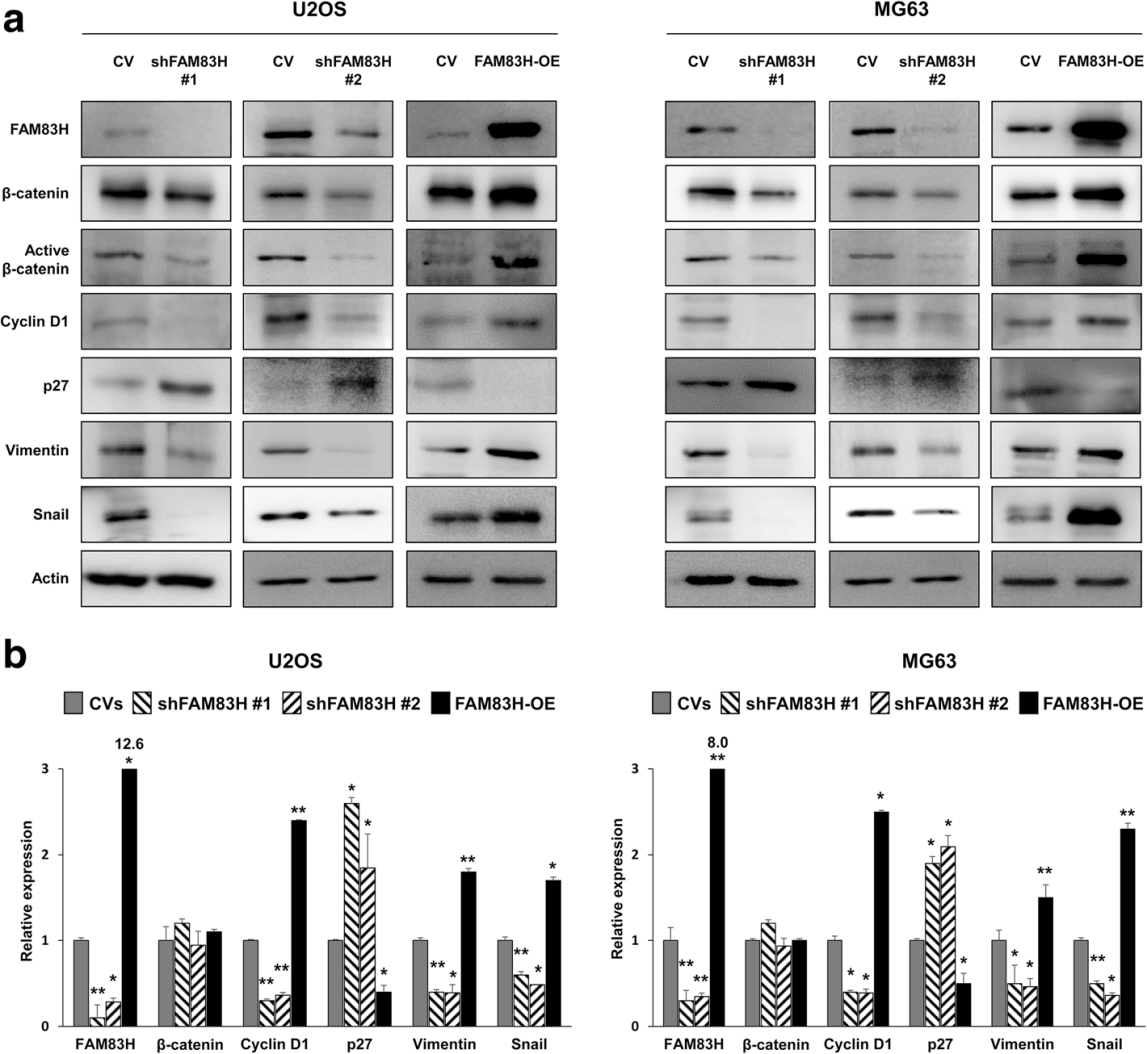
Western blotting and quantitative reverse-transcription polymerase chain reaction after knock-down or overexpression of FAM83H in osteosarcoma cells. **a** Western blotting for FAM83H, β-catenin, active β-catenin, cyclin D1, p27, vimentin, and snail after knock-down or overexpression of FAM83H in U2OS and MG63 osteosarcoma cells. **b** Quantitative reverse-transcription polymerase chain reaction for FAM83H, β-catenin, cyclin D1, p27, vimentin, and snail after knock-down or overexpression of FAM83H in U2OS and MG63 cells. CV; control vector, OE; overexpression, *; versus control vector transfection; *P <* 0.05, **; versus control vector transfection; *P <* 0.001

As the expression of FAM83H was significantly associated with advanced clinicopathological factors such as larger tumor size, higher tumor stage, and higher histologic grade, we evaluated the effect of the FAM83H on the proliferation and invasiveness of osteosarcoma cells. As expected, the knock-down of FAM83H with shRNA for FAM83H inhibited proliferation, and overexpression of FAM83H increased the proliferation of U2OS and MG63 osteosarcoma cells (Fig. 3a and b). In addition, the migration and invasion activities of osteosarcoma cells were significantly inhibited with knock-down of FAM83H and increased with overexpression of FAM83H in U2OS and MG63 cells (Fig. 3c and d). Moreover, overexpression of FAM83H significantly increased in vivo growth of KHOS/NP cells, and knock-down of FAM83H significantly inhibited in vivo growth of KHOS/NP cells (Fig. 4a and b). Furthermore, overexpression of FAM83H was significantly associated with increased pulmonary metastases (Fig. 4c). The five mice with FAM83H-overexpressing KHOS/NP cells showed grossly visible pulmonary metastatic nodules, but no grossly visible metastatic pulmonary nodule in neither cells transfected with control vectors nor shRNA for FAM83H. Microscopically, FAM83H-overexpressing cells showed more pulmonary metastasis compared with cells transfected with control vectors or shRNA for FAM83H (mean number of metastatic nodule per mice: FAM83H-overexpression; 9.4, control vectors; 1.6, shFAM83H; 0.8) (Fig. 4c). There was no metastasis in liver or kidney in all groups. In addition, FAM83H-related proliferation and invasiveness of osteosarcoma cells were related to the expression of β-catenin, cyclin D1, p27, snail, and vimentin. The expression of mRNA and protein of cyclin D1, snail, and vimentin were decreased with the knock-down of FAM83H and increased with the overexpression of FAM83H in both U2OS and MG63 cells (Fig. 5). The expression of p27 protein and mRNA were increased with knockdown of FAM83H and decreased with FAM83H overexpression (Fig. 5). However, despite no significant change in expression of mRNA of β-catenin with the knock-down or overexpression of FAM83H, the protein expression of β-catenin and active β-catenin were decreased with the knock-down of FAM83H and increased with the overexpression of FAM83H (Fig. 5).

### FAM83H is involved in stabilization of β-catenin

**Fig. 6:**
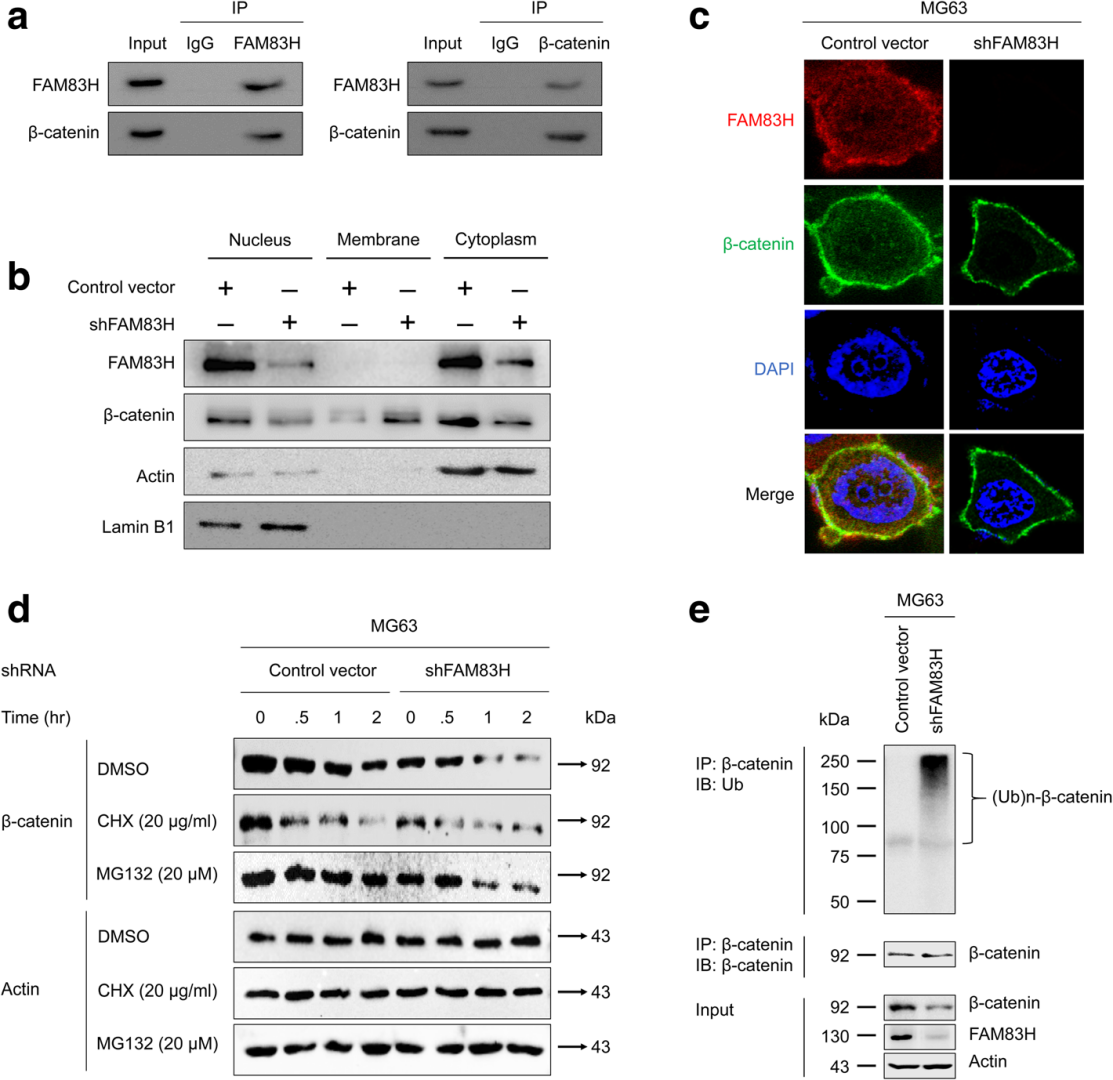
FAM83H directly bind to β-catenin and regulates the stabilization of β-catenin protein by the proteasome-mediated ubiquitination of β-catenin. **a** Immunoprecipitation for FAM83H and β-catenin. β-catenin was detected in the protein taken with immunoprecipitation for FAM83H and vice versa. **b** Western blot for FAM83H, β-catenin, actin, and lamin B1 with the protein lysates fractionated according to subcellular localization after transfection of shRNA for FAM83H in MG63 cells. **c** Confocal microscopic analysis with immunofluorescence staining for FAM83H (red) and β-catenin (green) after knock-down of FAM83H with shRNA for FAM83H in MG63 cells. The co-localization of FAM83H and β-catenin is colored yellow. **d** The MG63 cells were transfected with control vector or shRNA for FAM83H. 48 h after transfection, the cells were treated with 20 μg/ml cycloheximide or 20 μM MG132 for 0.5 to 2 h. The cell lysates were blotted with anti-β-catenin antibody and anti-β-actin antibodies. **e** The MG63 cells were transfected with control vector or shRNA for FAM83H for 48 h. Subsequently, the transfected cells were treated with MG132 (20 μM) for 4 h and total lysates of cells were immunoprecipitated with anti-β-catenin antibodies and blotted with anti-ubiquitin and anti-β-catenin antibodies

As we have shown in Fig. 5, FAM83H did not affect the mRNA expression of β-catenin, but the expression of total β-catenin and active β-catenin were associated with FAM83H expression. Therefore, we further evaluated the relationship between FAM83H and β-catenin. Immunoprecipitation for FAM83H or β-catenin in MG63 cells showed complex formation of FAM83H and β-catenin. After immunoprecipitation with FAM83H, immunoblot bands for β-catenin was detected and vice versa (Fig. 6a). In addition, when we performed fractionation of cell lysates of MG63 cells after knock-down of FAM83H, cytoplasmic and nuclear expression of β-catenin was low compared with the cells transfected with control shRNA (Fig. 6b). The confocal microscopic analysis also showed weak expression of β-catenin in the cytoplasm and nuclei of MG63 cells, which were subjected to a knock-downed FAM83H (Fig. 6c). Because FAM83H influenced the expression and subcellular localization of β-catenin, we investigated whether FAM83H is involved in post-translational protein stabilization of β-catenin. For this purpose, we examined the protein stability of β-catenin in MG63 cells via treatment with cycloheximide after transfection with either control shRNA or FAM83H shRNA. As shown in Fig. 5d, the protein stability of β-catenin in MG63 cells transfected with FAM83H shRNA was greatly reduced (as indicated by more rapid depletion) than in MG63 cells transfected with control shRNA. Furthermore, the protein expression of β-catenin in MG63 cells transfected with FAM83H shRNA decreased within one-hour of treatment of MG132, but the protein expression of β-catenin in MG63 cells transfected with control shRNA did not decrease after up to two hours (Fig. 6d). These results suggest that the decreased expression level of β-catenin with knock-down of FAM83H depends on proteasome-mediated protein degradation. Subsequently, we assessed whether ubiquitination of β-catenin is involved in the proteasome-mediated β-catenin degradation. MG63 cells were transfected with control or FAM83H shRNA and incubated with MG132 for four hours. Then, total protein lysates were immunoprecipitated with β-catenin antibody and immunoblotted with anti-ubiquitin antibody. As shown in Fig. 6e, immunoprecipitated β-catenin MG63 cells transfected with FAM83H shRNA clearly showed the poly-ubiquitination pattern of β-catenin compared to MG63 cells transfected with control shRNA.

## Discussion

In this study, we investigate the roles and relationship of FAM83H and β-catenin in osteosarcomas and show that the expression of FAM83H and β-catenin are closely associated, and their expression are involved in the progression of osteosarcomas. In human osteosarcoma tissue samples, the expression of Cy-FAM83H and Nu-FAM83H were associated with advanced clinicopathologic factors such as larger tumor size, higher tumor stage, and higher histologic grade of osteosarcomas. Moreover, both Cy-FAM83H and Nu-FAM83H positivity were significantly associated with shorter OS and RFS of osteosarcoma patients by univariate analysis. Despite the limited number of cases of osteosarcomas, higher expression of Cy-FAM83H was an independent indicator of poor prognosis and shorter OS and RFS of osteosarcoma patients in multivariate analysis. In line with these results, higher expression of FAM83H was significantly associated with higher tumor stage and shorter survival of hepatocellular carcinomas and clear cell renal cell carcinoma patients [4, 10]. In addition, the expression of FAM83H gene was consistently overexpressed in various human cancers such as lung, breast, colon, liver, ovary, pancreas, and stomach cancers, and higher expression of FAM83H gene was associated with shorter survival of uterine cancer patients [8]. However, in contrast, gene expression of FAM83H lower in brain astrocytoma and brain oligodendroglioma, and lower expression of FAM83H in these tumors was associated with shorter disease-free survival of patients [8]. Therefore, there is a possibility that the role of FAM83H in tumorigenesis might differ according to cancer types. However, studies on the role of FAM83H in human cancers and the number of cases of osteosarcoma analyzed in this study are limited. Therefore, further study specifically focused on the clinical significance of FAM83H expression in human cancers is needed. However, despite a difference in the prognostic role of FAM83H according to the type of cancer and the limited number of cases of osteosarcoma used in this study, our results suggest that FAM83H expression might be used as a prognostic marker for osteosarcoma patients.

Poor prognosis in osteosarcoma patients with tumors with elevated expression of FAM83H suggests that FAM83H is involved in the progression of osteosarcomas. Supportively, in this study, in addition to the prognostic significance of Cy-FAM83H and Nu-FAM83H expression in osteosarcoma patients, overexpression of FAM83H increased proliferation of osteosarcoma cells, and knock-down of FAM83H inhibited proliferation of osteosarcoma cells both in vitro and in vivo. Moreover, overexpression of FAM83H increased expression of β-catenin, active β-catenin, and cyclin D1, and decreased expression of p27. In line with our results, knock-down of FAM83H inhibited proliferation of prostate cancer cells [9] and clear cell renal cell carcinoma cells [10]. In hepatocellular carcinomas, overexpression of FAM83H increased proliferation of cancer cells in vitro and in vivo [4]. These findings suggest that FAM83H plays a key role in the proliferation of cancer cells. In addition to the role of FAM83H in the proliferation of cancer cells, FAM83H was involved in the invasiveness of cancer cells. Overexpression of FAM83H increased the migration and invasion activity of osteosarcoma cells, which was associated with increased expression of vimentin and snail. Moreover, overexpression of FAM83H was associated with more pulmonary metastasis of KHOS/NP cells in vivo (Fig. 4c). With respect to cancer progression, the expression of vimentin and snail is are characteristic of the epithelial-to-mesenchymal transition (EMT) [30, 31]. Especially, snail is an important transcription factor driving EMT [32, 33] and is involved in the invasiveness of cancer cells [34]. In human breast cancers, the expression of snail was associated with shorter survival of patients [35]. Therefore, when considering the roles of FAM83H in the expression of vimentin and snail, FAM83H-mediated EMT, which enhances invasiveness of osteosarcomas, might explain how FAM83H is involved in the progression of osteosarcoma. Consistently, knock-down of FAM83H disrupted keratin cytoskeleton organization and inhibited the migration activity of colon cancer cells [5]. In hepatocellular carcinoma cells, snail expression was associated with FAM83H expression-mediated invasiveness [4]. However, in contrast to carcinoma cells, sarcoma cells are mesenchymal cells basically expressing vimentin. Therefore, the expression of vimentin and snail might not be sufficient to explain the invasiveness of sarcoma cells and the concept of EMT in osteosarcoma poses a paradox [32]. However, although the underlying biologic mechanism of EMT in sarcoma is not precise, EMT has been suggested as an important factor in sarcoma progression [32]. Therefore, when considering the expression of vimentin and snail as markers of EMT [32, 33], FAM83H-mediated expression of vimentin and snail might explain how FAM83H is involved in the invasiveness of osteosarcoma cells.

Another interesting finding of this study is that there was a significant correlation between FAM83H expression and β-catenin expression in human osteosarcoma tissue samples. Especially, the expressions of both FAM83H and β-catenin were associated with shorter survival of osteosarcoma patients. These findings suggest that both FAM83H and β-catenin are closely associated and involved in the progression of osteosarcomas. The Wnt/β-catenin signaling is important in the proliferation and progression of human cancer cells [36–39]. Maintaining intracellular levels of β-catenin is one of the important processes in neoplastic cells to maintain sustained proliferation of cancer cells [36–38]. Therefore, the mechanism involved in the stabilization of β-catenin is vital in development and progression of tumors. In this study, we presented a mechanism of β-catenin stabilization in osteosarcoma cells. In MG63 osteosarcoma cells, knock-down or overexpression of FAM83H did not significantly affect the level of β-catenin mRNA, but the protein level of β-catenin decreased with knock-down of FAM83H and increased with overexpression of FAM83H. In addition, FAM83H directly bound to β-catenin and retarded proteasomal degradation of β-catenin. When we induced knock-down of FAM83H, ubiquitination of β-catenin increased which subsequently increased its degradation. Therefore, FAM83H is important in the sustained expression of β-catenin in osteosarcoma cells. Consistently, FAM83H-mediated stabilization of β-catenin has been suggested in colorectal cancers [40]. Overexpression of FAM83H suppressed cytoplasmic CK1α and thereby supported nuclear localization of β-catenin with de-phosphorylation mediated stabilization [40]. In liver and kidney cancer, FAM83H expression was associated with the expression of cyclin D1, cyclin E1, and downstream signaling of the Wnt/β-catenin pathway [4, 10]. Therefore, these findings suggest that FAM83H is involved in the progression of osteosarcoma by mediating stabilization of β-catenin from its proteasomal degradation. Therefore, our result suggests that the FAM83H-β-catenin pathway might be a new therapeutic target of osteosarcomas. In addition, in our results, the expression of β-catenin was significantly associated with shorter OS and RFS of osteosarcoma patients in univariate analysis. Although there is one controversial report [41], the expression of β-catenin has been reported to be associated with poor prognosis of cancer patients including those with soft-tissue sarcomas [21]. Therefore, the expression of β-catenin is might also be used as a prognostic marker for osteosarcomas.

Concerning the subcellular localization of FAM83H, FAM83H was considered a cytoplasmic membranous protein involved in the cytoskeletal arrangement [5, 6, 29]. However, as we have shown in human osteosarcoma tissue samples, western blotting of subcellular fractionated protein and confocal microscopic images indicate that FAM83H expression was observed in the cytoplasmic membrane, cytoplasm, and nuclei. In addition, both Cy-FAM83H and Nu-FAM83H expression were significantly associated with higher stage, larger tumor size, and higher histologic grade of osteosarcomas. Moreover, both Cy-FAM83H and Nu-FAM83H expression was significantly associated with shorter survival of osteosarcoma patients in univariate analysis. Consistently, both Cy-FAM83H and Nu-FAM83H expression were associated with shorter survival of hepatocellular carcinoma and clear cell renal cell carcinoma patients [4, 10]. In multivariate analysis, Nu-FAM83H expression was an independent indicator of shorter survival of hepatocellular carcinoma and clear cell renal cell carcinoma patients [4, 10]. Therefore, nuclear localization of FAM83H was suggested as an important indicator of cancer progression. However, in osteosarcoma, the statistical prognostic significance of Cy-FAM83H expression was potent in comparison to Nu-FAM83H expression in multivariate analysis. This discrepancy might be related to the relatively small population of osteosarcomas evaluated in this study. Therefore, further study is needed to clarify the role of FAM83H depending on its subcellular localization. However, despite this limitation, knock-down or overexpression of FAM83H influenced proliferation and/or invasiveness of various human cancers such as osteosarcoma, prostate cancer, hepatocellular carcinoma, and clear cell renal cell carcinoma patients [4, 9, 10]. Therefore, it is suggested that the overall expression of FAM83H is important in the progression of FAM83H and higher expression of FAM83H might promote tumor progression.

## Conclusions

In conclusion, we showed that FAM38H expression is important for the proliferation and invasiveness of osteosarcoma cells and suggest that stabilizing β-catenin protein by FAM38H can be a biochemical mechanism to explain the significant association between FAM38H expression and shorter survival of osteosarcoma patients. These results highlight FAM83H as a new prognostic maker as well as a new therapeutic target of osteosarcoma.

## Data Availability

The datasets used in the current study are available from the corresponding author upon reasonable request.

## Abbreviations

95% CI: (95% confidence interval)
Cy-FAM83H: (Cytoplasmic expression of FAM83H)
HR: (hazard ratio)
MTT: (3-(4,5-dimethylthiazol-2-yl)-2,5-iphenyltetrazolium bromide)
Nu-FAM83H: (nuclear expression of FAM83H)
OS: (overall survival)
RFS: (relapse-free survival)
TMA: (The tissue microarray)
